# Proteomics of the astrocyte secretome reveals changes in their response to soluble oligomeric Aβ

**DOI:** 10.1111/jnc.15875

**Published:** 2023-06-11

**Authors:** Vittoria Matafora, Alena Gorb, Fangjia Yang, Wendy Noble, Angela Bachi, Beatriz Gomez Perez‐Nievas, Maria Jimenez‐Sanchez

**Affiliations:** ^1^ IFOM ETS‐ The AIRC Institute of Molecular Oncology Milan Italy; ^2^ Department of Basic and Clinical Neuroscience Maurice Wohl Clinical Neuroscience Institute, Institute of Psychiatry, Psychology and Neuroscience, King's College London London UK

## Abstract

Astrocytes associate with amyloid plaques in Alzheimer's disease (AD). Astrocytes react to changes in the brain environment, including increasing concentrations of amyloid‐β (Aβ). However, the precise response of astrocytes to soluble small Aβ oligomers at concentrations similar to those present in the human brain has not been addressed. In this study, we exposed astrocytes to media from neurons that express the human amyloid precursor protein (*APP)* transgene with the double Swedish mutation (APPSwe), and which contains APP‐derived fragments, including soluble human Aβ oligomers. We then used proteomics to investigate changes in the astrocyte secretome. Our data show dysregulated secretion of astrocytic proteins involved in the extracellular matrix and cytoskeletal organization and increase secretion of proteins involved in oxidative stress responses and those with chaperone activity. Several of these proteins have been identified in previous transcriptomic and proteomic studies using brain tissue from human AD and cerebrospinal fluid (CSF). Our work highlights the relevance of studying astrocyte secretion to understand the brain response to AD pathology and the potential use of these proteins as biomarkers for the disease.

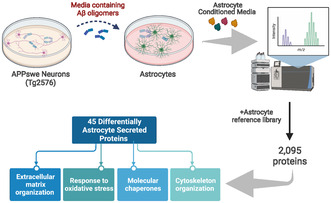

## Graphical abstract

Astrocytes react to Aβ plaques in AD, however, the precise response of astrocytes to amyloid‐β (Aβ) remains uncertain. Here, we undertook a proteomic analysis of the astrocyte secretome in response to media containing Aβ oligomers. This study revealed differential secretion of proteins involved in the extracellular matrix and cytoskeletal organization, changes in the response to oxidative stress, and increased secretion of proteins with chaperone activity.

**Abbreviations**
AD: Alzheimer's disease
APP: amyloid precursor protein
APPSwe_ACM: conditioned media from astrocytes treated with APPSwe_NCM
APPSwe_NCM: conditioned media from neurons from Tg2576 mice
Aβ: beta‐amyloid
CSF: cerebrospinal fluid
ECM: extracellular matrix
GFAP: glial fibrillary acidic protein
GO: gene ontology
MGI: mouse genome informatics
MS: mass spectrometry
RRID: Research Resource Identifier
WT_ACM: conditioned media from astrocytes treated with WT_NCM
WT_NCM: conditioned media from neurons from wild‐type mice

## 1 INTRODUCTION

Alzheimer's disease (AD) pathology is classically defined by the extracellular deposition of amyloid‐β (Aβ) and the intracellular accumulation of hyperphosphorylated tau, which is accompanied by neuronal and synapse loss, leading to cognitive impairment (Knopman et al., 2021). Astrocyte reactivity, involving transcriptional, morphological, and functional changes in astrocytes in response to Aβ and tau, is also a key feature of AD (Escartin et al., 2021; Perez‐Nievas & Serrano‐Pozo, 2018). Glial fibrillary acidic protein (GFAP)‐positive astrocytes usually cluster around amyloid plaques (Itagaki et al., 1989; Serrano‐Pozo et al., 2013). Astrocyte reactivity indicated by increased GFAP expression is one of the characteristics that allow AD‐demented cases to be distinguished from those who are clinically non‐demented while having AD‐like pathology (Barroeta‐Espar et al., 2019; Perez‐Nievas et al., 2013), and evidence suggests that this astrocyte reaction precedes Aβ and tau deposition in the human brain (Carter et al., 2012; Rodriguez‐Vieitez et al., 2016). Recently, plasma GFAP levels have been associated with increased Aβ pathology (Benedet et al., 2021; Pereira et al., 2021) and are elevated in older individuals at risk of AD (Chatterjee et al., 2021) further highlighting the relevance of astrocytes in the disease.

Astrocytes react to Aβ plaques in AD, however, the specific functional changes that this reactivity causes and the implications in the onset and progression of AD remain uncertain. Exposure to extracellular Aβ induces an astrocytic response in culture, as evidenced by an increase in astrocyte‐mediated neurotoxicity through the release of N‐SMase (Jana & Pahan, 2010) and soluble inflammatory factors (Garwood et al., 2011), or increased synaptotoxicity mediated by the secretion of factors such as glutamate (Talantova et al., 2013), complement C3 (Lian et al., 2015, 2016), or CXCL1 (Perez‐Nievas et al., 2021). Additionally, Aβ exposure may interfere with astrocytic protective functions as it results in decreased secretion of synaptogenic factors such as TSP‐1 (Rama Rao et al., 2013) or TGF‐β1 (Diniz et al., 2017) and impairs phagocytosis and degradation of dystrophic neurites (Sanchez‐Mico et al., 2021).

Previous proteomics studies have characterized the astrocyte secretome in resting conditions (Dowell et al., 2009; Greco et al., 2010; Han et al., 2014; Lafon‐Cazal et al., 2003) and in response to proinflammatory stimuli such as lipopolysaccharide (LPS) or cytokines (Delcourt et al., 2005; Keene et al., 2009; Lafon‐Cazal et al., 2003), as well as specific insults including cholinergic stimulation (Moore et al., 2009), angiogenin (Skorupa et al., 2013), mechanic injury (Lai et al., 2013; Thorsell et al., 2008) or in response to endoplasmic reticulum (ER) stressors (Smith et al., 2020). The astrocyte secretome has also been characterized in response to synthetic Aβ42 (Lai et al., 2013). However, much of the existing research characterizing the astrocyte response in AD is limited due to the lack of appropriate tools to mimic in culture the species and concentrations of Aβ found in the AD brain. Synthetic Aβ peptides have been typically used at doses that exceed the concentrations of Aβ found in AD brain by 100–1000 times (Allaman et al., 2010; Diniz et al., 2017; Lai et al., 2013; Varshavskaya et al., 2022). In addition, the nature of the peptides is crucial to determine their underlying effects. Rather than monomers or highly aggregated forms, soluble Aβ oligomers are the species typically associated with synaptic dysfunction and loss of dendritic spines (Arbel‐Ornath et al., 2017; Lacor et al., 2004; Shankar et al., 2007, 2008), increased tau phosphorylation and missorting into dendrites (Zempel et al., 2010), impairment of axonal transport (Sherman et al., 2016; Vossel et al., 2010) or increased reactive oxygen species (ROS) production (Behl et al., 1994). Investigating how astrocyte secretion is modulated in response to naturally secreted forms of Aβ will better help to understand the contribution of astrocytes to AD pathology.

In this study, we characterized changes in the astrocyte secretome in response to soluble Aβ oligomers, at concentrations and species similar to those present in the human brain, secreted into the culture medium from neurons that express a human *APP* transgene with the double Swedish mutation (APPSwe, Tg2576 mice (Hsiao et al., 1996)). APPSwe neurons also secrete other human APP‐derived fragments (Wu et al., 2010), providing a cellular environment that resembles that one experienced in the brain. Using an optimized methodology to analyze the extracellular media with mass spectrometry while discriminating serum components, we generated a list of proteins whose levels are significantly changed in the astrocyte secretome upon treatment with neuron‐derived Aβ oligomers. We used functional and pathway annotation to explore the processes that are altered in astrocytes in these disease‐mimicking conditions. Our data identified that Aβ exposed astrocytes show altered secretion of proteins involved in the reorganization of the extracellular matrix and the cytoskeleton, as well as in protective antioxidant and chaperone function responses. Some of the identified proteins are altered in CSF and tissues from AD patients, supporting the idea that astrocyte‐secreted proteins can be explored as potential biomarkers for disease and can aid the understanding of functional astrocyte changes in AD.

## 2 METHODS

### 2.1 Animals

All animal work was conducted in accordance with the UK Animals (Scientific Procedures) Act 1986 and the European Directive 2010/63/EU under UK Home Office Personal and Project Licenses and with agreement from the King's College London (Denmark Hill) Animal Welfare and Ethical Review Board. No specific Ethics Approval number was mandated. Pregnant female CD1 mice (MGI:5649524) purchased from Charles River were used to prepare mouse primary astrocyte cultures. Tg2576 mice (MGI:2385631) were used to prepare primary neurons that secrete human Aβ. Tg2576 mice overexpress human APP (isoform 695) containing the double mutation K670N, M671L (Swedish mutation) under the control of the hamster prion protein promoter (Hsiao et al., 1996). Tg2576 mice were originally obtained from Taconic farms (Germantown, NY, USA), and were maintained in‐house by breeding males with C57Bl/6/SJL F1 (MGI:5655343) females as recommended by the suppliers to minimize aggressive phenotypes. Moderate severity phenotypes were monitored, and animals were bred before 6 months of age. Up to 5 animals of the same gender were kept in the same cage. The genotype of the animals was determined by polymerase chain reaction on DNA obtained from the embryos, as previously described (Mitchell et al., 2009). Water and food were available (Picolab rodent diet 20; # 5053; Lab Diet) ad libitum. Animals were housed at 19–22°C, humidity 55%, 12‐h:12‐h light:dark cycle with lights on at 07:30. A total of 5 CD1 dams were used to obtain pools of 10 pups on average that were used to prepare primary glial cultures. A total of 5 breeding pairs from Tg2576 and C57Bl/6/SJL crosses were used to obtain embryos from which to prepare either wild type or Tg2576 neuronal cultures. No animals were excluded. Mice were sacrificed by cervical dislocation or decapitation according to Home Office‐approved protocol.

### 2.2 Culture of primary cortical neurons and collection of neuronal conditioned media

To obtain oligomeric Aβ enriched conditioned media, we cultured neurons from the cerebral cortex of Tg2576 mice bearing the human APPSwe mutation at embryonic day 15 (E15) as previously described (DaRocha‐Souto et al., 2012; Perez‐Nievas et al., 2021; Wu et al., 2010). Primary cortical neurons were isolated from each E16.5 embryo resulting from the mating between a *Tg2576* male that heterozygously overexpresses a human mutated *APP* gene and a wild‐type female, thus giving rise to both transgenic (APPswe) and littermate (WT) cultures. The expression of the APPswe gene was determined for each embryo. Briefly, brains were harvested and placed in ice‐cold HBSS with HEPES, where the meninges were removed, and the cerebral cortices were dissected and digested in TryplE (Gibco™, cat no. 12605010). Cells were cultured in polyD‐lysine (Gibco™, cat no. P7280) coated plates and grown in Neurobasal medium (Gibco™, cat no. 21103049) supplemented with 2% B27 (Gibco™, cat no. 17504044), GlutaMAX (Gibco™, cat no. 35050061), sodium pyruvate (Gibco™, cat no. 11360070), and 100 units/mL penicillin and 100 μg/mL streptomycin (Gibco™, cat no. 15140122) at 37°C in a humidified incubator with 5% CO_2_. After 14 DIV, media from transgenic mice (APPSwe_NCM) and wild‐type control (WT_NCM) was collected. The concentration of Aβ in the media was determined with an ELISA kit (LifeTechnologies, cat no. KHB3481 and KHB3441). The concentration of Aβ40 and Aβ42 in the neuron media was adjusted to 2000 and 200 pM, respectively, in Neurobasal media with 0.5% B27, to limit the amount of high‐abundance proteins such as albumin. Equivalent amounts were used to prepare media from control neurons (WT_NCM).

### 2.3 Culture of primary mouse astrocytes and collection of astrocyte lysates and conditioned media

Primary astrocyte cultures were prepared from the cortex of wild‐type CD1 mice on postnatal days 1–3 as previously described (Schildge et al., 2013). Briefly, brains from a littermate were harvested and placed in ice‐cold HBSS with HEPES, where the meninges were removed, and the cerebral cortices were dissected, pooled together, and mechanically dissociated. Mixed glial cells were cultured in poly‐D‐lysine coated flasks and grown in high glucose (Gibco™, cat no. DMEM 21969035) media with 10% fetal bovine serum (Gibco™, cat no. 10500064), GlutaMAX™ supplement, and 100 units/mL penicillin and 100 μg/mL streptomycin at 37°C in a humidified incubator with 5% CO_2_. Media was changed after 2 days and then every 5 days. On days 11–14, microglia cells and oligodendrocytes were removed by overnight shaking at 200 rpm and astrocytes were seeded on poly‐D‐lysine (Sigma, cat no. P6407) coated 6‐multiwell plates and were used after 3 days at 80–90% confluency (>95% of cells were GFAP‐positive).

Astrocyte growth medium was washed and changed to Neurobasal media supplemented with 0.5% B27 medium 24 h prior to treatment. Astrocytes were cultured in Neurobasal media without B27 for 24 h, to collect astrocyte‐conditioned media to prepare an in‐house library. Astrocytes were cultured in APPswe_NCM and WT_NCM for 24 h, to collect astrocyte conditioned media to analyze by mass spectrometry (APPse_ACM and WT_ACM). Media were filtered through a 0.22 μm spin filter to eliminate any detached cells or debris and concentrated through a 3 kDa molecular weight cut‐off concentrating column (Millipore, cat no. UFC500396) at 13 000 *g* for 30 min, prior to analysis. For validation by western blotting, cells were washed once in PBS and harvested on lysis buffer (20 mM Tris–HCl pH 6.8, 137 nM NaCl, 1 mM EGTA, 1% Triton X‐100, 10% glycerol, 1x Roche complete mini protease inhibitor).

### 2.4 Secretome analysis

Conditioned media from 5 independent neuronal cultures, with each biological replicate analyzed in duplicate, were analyzed as previously described (Matafora et al., 2020) by Secret3D workflow (Matafora & Bachi, 2020). Collected media was filtered on microcon filters with 10 KDa cutoff (Millipore, cat no. MRCPRT010), and buffer was exchanged with 8 M Urea 100 mM Tris pH 8. 50 mg of secreted protein was sonicated with BIORUPTOR (3 cycles: 30 s on/30 s off). Cysteine reduction and alkylation were performed by adding 10 mM TCEP (Thermo Scientific, cat no. 20490) and 40 mM 2‐chloroacetamide (Sigma‐Aldrich, cat no. C0267) in 8 M Urea 100 mM Tris pH 8 for 30 min at room temperature. By using microcon filters with 10 kDa cutoff (Millipore), the buffer was exchanged by centrifugation at 9300 *g* for 10 min, and PNGase F (New England Biolabs, cat no. P0708) (1:100 = enzyme: secreted proteins) was added for 1 h at room temperature following manufacturer's instruction. Buffer was again exchanged by centrifugation at 9300 g for 10 min with 50 mM ammonium bicarbonate, and proteins in the solution were digested by Lys‐C and trypsin (Kulak et al., 2014). Peptides were recovered on the bottom of the microcon filters by centrifugation at 9300 g for 10 min, adding two consecutive washes of 50 μL of 0.5 M NaCl. Eluted peptides were purified on a homemade C18 StageTip. 1 μg of the digested sample was injected onto a quadrupole Orbitrap Q‐exactive HF mass spectrometer (Thermo Scientific). Peptide separation was achieved on a linear gradient from 95% solvent A (2% ACN, 0.1% formic acid) to 55% solvent B (80% acetonitrile, 0.1% formic acid) over 75 min and from 55% to 100% solvent B in 3 min at a constant flow rate of 0.25 μL/min on UHPLC Easy‐nLC 1000 (Thermo Scientific) where the LC system was connected to a 23‐cm fused‐silica emitter of 75 μm inner diameter (New Objective, Inc.), packed in‐house with ReproSil‐Pur C18‐AQ 1.9 μm beads (Dr. Maisch Gmbh, Ammerbuch, Germany) using a high‐pressure bomb loader (Proxeon).

The mass spectrometer was operated in DDA (Data Dependent Acquisition) mode: dynamic exclusion enabled (exclusion duration = 15 s), MS1 resolution = 70 000, MS1 automatic gain control target = 3 × 106, MS1 maximum fill time = 60 ms, MS2 resolution = 17 500, MS2 automatic gain control target = 1 × 105, MS2 maximum fill time = 60 ms, and MS2 normalized collision energy = 25. For each cycle, one full MS1 scan range = 300–1650 m/z was followed by 12 MS2 scans using an isolation window of 2.0 m/z.

The mass spectrometry proteomics data have been deposited to the ProteomeXchange Consortium via the PRIDE (www.ebi.ac.uk/pride/) (Perez‐Riverol et al., 2022) partner repository with the dataset identifier PXD036343.

### 2.5 MS analysis and database search

MS analysis was performed as reported previously (Geyer et al., 2016). Raw MS files were processed with MaxQuant software (1.6.0.16), making use of the Andromeda search engine (Cox et al., 2011). MS/MS peak lists were searched against the UniProtKB Mouse complete proteome database (uniprot_cp_mouse_2019) in which trypsin specificity was used with up to two missed cleavages allowed. Searches were performed selecting alkylation of cysteine by carbamidomethylation as fixed modification, and oxidation of methionine, N‐terminal acetylation, and N‐Deamination as variable modifications. Mass tolerance was set to 5 ppm and 10 ppm for parent and fragment ions, respectively. A reverse decoy database was generated within Andromeda, and the false discovery rate (FDR) was set to <0.01 for peptide spectrum matches (PSMs). For identification, at least two peptide identifications per protein were required, of which at least one peptide had to be unique to the protein group. Matching between runs was performed across all samples that are neuron‐conditioned media and astrocyte‐conditioned media, plus conditioned media from astrocyte without serum that was used as reference library.

### 2.6 ELISA

A quantitative ELISA kit was used to detect mouse APOE (Abcam, Cat no. Ab215086) in conditioned media according to the manufacturer's instructions.

### 2.7 Western blotting

Media concentrated through a 3 kDa molecular weight cut‐off column and cell lysates were resuspended and boiled in Laemmli buffer. Samples were subjected to 12% SDS‐PAGE and transferred to a nitrocellulose membrane (Amersham Protran Cat no. 10600002). After blocking in Odyssey Blocking buffer (Li‐Cor, Cat no. 927‐600001), blots were probed overnight with primary antibody: anti‐PPIB (1:1000; Thermo cat no. PA1‐027A) or anti‐GAPDH (1:1000: Santa Cruz, Cat no. sc‐32233), followed by 2 h incubation in the appropriate anti‐mouse or anti‐rabbit secondary antibodies. Signal was visualized using an Odyssey CLx infrared imager (LI‐COR Biosciences) and quantified using ImageStudio Lite (Li‐COR) software.

### 2.8 Quantification and statistical analysis

Sample size was estimated based on previous analysis where changes in astrocyte secretion were determined in response to similar media containing Aβ oligomers (Perez‐Nievas et al., 2021) and secretome studies and was further validated by principal component analysis (PCA). Blinding was not performed, sample collection and mass spectrometry were performed by different experimenters and at different facilities. DDA.raw files were analyzed by MaxQuant software for protein quantitation, and LFQ intensities were used. Statistical analysis was performed by using Perseus software (version 1.5.6.0) included in the MaxQuant package. Outliers were excluded based on the Principal Component Analysis (PCA) of all the samples. Assessment of the normality of data was carried out by using Perseus software. Histogram of all the values log2 transformed was performed to confirm if the transformed matrix follows the normal distribution prior to T‐test statistical analysis that was performed applying FDR <0.05 or *p* < 0.05, as reported.

### 2.9 Protein localization prediction

SignalP‐6.0 (https://services.healthtech.dtu.dk/service.php?SignalP) (Teufel et al., 2022), TargetP‐2.0 (https://services.healthtech.dtu.dk/service.php?TargetP‐2.0) (Almagro Armenteros et al., 2019), SecretomeP‐2.0 (https://services.healthtech.dtu.dk/service.php?SecretomeP‐2.0) (Bendtsen et al., 2004), DeepLoc‐2.0 (https://services.healthtech.dtu.dk/service.php?DeepLoc‐2.0) (Thumuluri et al., 2022), ProtComp 9.0 (http://www.softberry.com/berry.phtml?topic=protcompan&group=programs&subgroup=proloc) (Softberry, Inc.), and GO annotations from LAGO analysis were used to identify which proteins are predicted to be secreted or found in the extracellular space. DeepTMHMM (https://dtu.biolib.com/DeepTMHMM) (Hallgren et al., 2022) was used to predict the presence of transmembrane helices, and the Vesiclepedia database (http://microvesicles.org/) (Pathan et al., 2019) was used for the manual search of proteins reported to be secreted via extracellular vesicles.

### 2.10 Bioinformatics analysis

Gene ontology (GO) term over‐representation for biological process, molecular function, and cell component were identified using LAGO (Boyle et al., 2004) (https://go.princeton.edu/). P‐values were calculated based on the hypergeometric distribution with the Bonferroni correction for multiple comparisons, with *p* < 0.05 after the correction considered significant. STRING 11.5 (https://string‐db.org/) (Szklarczyk et al., 2021) was used to predict protein–protein interactions (PPIs) within the differentially expressed proteins, and local STRING network clusters were used to identify if any of the interacting proteins take part in the same biological process. Pathway enrichment analysis was performed using the Reactome pathway database using the online PANTHER v16.0 software (http://pantherdb.org/) (Mi et al., 2021) using Fisher's Exact test and FDR to correct for multiple comparisons. Statistical significance was defined as FDR <0.05. Functional Annotation Clustering of differentially upregulated proteins was performed using the Database for Annotation, Visualisation, and Integration Discovery (DAVID, (Sherman et al., 2022; https://david.ncifcrf.gov/home.jsp)). Terms from Gene Ontology (GOTERM_BP_DIRECT, GOTERM_CC_DIRECT, and GOTERM_MF_DIRECT) and Pathways (KEGG_PATHWAY, REACTOME_PATHWAY, and WIKIPATHWAYS) were included in clustering analysis. The Enrichment Threshold EASE score was set at 1.3 (*p*‐value ≤0.05). Classification stringency was set at high. In line with the analysis performed with LAGO and Reactome, the whole genome from Mus musculus was used as a background. Gene‐disease Association (GDA) scores of genes for the differentially expressed proteins were calculated using DisGeNET v7.0 (https://www.disgenet.org/) (Piñero et al., 2021). The genes were filtered for association with the following terms: ‘Disease: Alzheimer's Disease; CUI: C0002395’ and ‘Disease: Familial Alzheimer Disease (FAD); CUI: C0276496’.

## 3 RESULTS

### 3.1 Analysis of the astrocyte secretome using an in‐house library shows changes in response to Aβ oligomers

**FIGURE 1:**
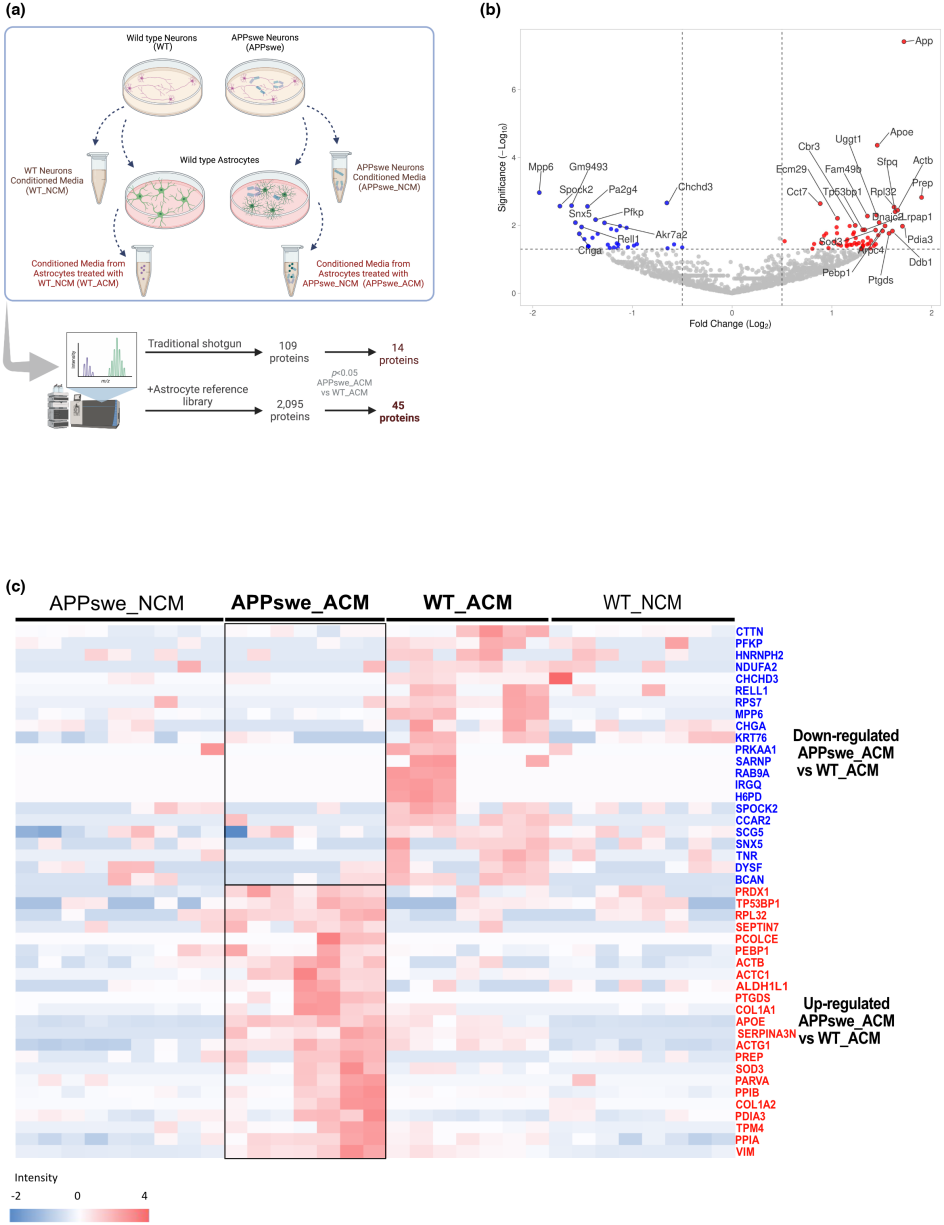
Proteomics analysis of the astrocyte conditioned media in response to Aβ‐containing media. (a) Schematic representation of the experimental approach. Conditioned media (NCM) from primary neurons prepared from Tg2576 mice (APPswe) or wild‐type littermates (WT) mice were collected at DIV14. Wild‐type astrocytes were treated with either APPswe‐conditioned media (APPswe_NCM) or WT‐conditioned media (WT_NCM), and the resultant astrocyte‐conditioned media (ACM) was analyzed by mass spectrometry. Traditional shotgun proteomics identified 109 proteins differentially secreted by astrocytes treated with Aβ‐containing media while referencing to an astrocyte library increased the yield to 2095 proteins. Created with BioRender.com. (b) Volcano plot showing proteins significantly up (red) and downregulated (blue) in the secretome of Aβ‐stimulated astrocytes, APPswe_ACM, compared to control, WT_ACM, before proteins contained in neuron media were excluded. The significance threshold was set at ≥1.3 and the absolute fold change threshold was set at >0.5. (c) Heatmap of proteins differentially secreted from Aβ‐stimulated astrocytes, after excluding proeins present in NCM. The blue‐to‐red scale indicates low‐to‐high protein levels. Differentially expressed proteins were defined based on a *p* < 0.05.

To investigate changes in the astrocyte secretome in response to soluble Aβ oligomers similar to those found in human disease brain (Arbel‐Ornath et al., 2017; Shankar et al., 2008; Wu et al., 2010), we exposed primary mouse astrocytes to conditioned media of neurons derived from Tg2576 mouse embryos that express a human *APP* transgene harboring the double Swedish mutation K670N, M671L (APPSwe, Hsiao et al., 1996) or from their wild type littermates (WT) (Figure 1a). The presence of low molecular weight Aβ oligomers in Tg2576 media has been widely characterized (DaRocha‐Souto et al., 2012; Perez‐Nievas et al., 2021; Wu et al., 2010), with Aβ42:Aβ40 ratios of 1:10 as in human AD brain, and concentrations ranging between 2 and 8 nM, similar to what it has been reported in human CSF (Snider et al., 2009). Conditioned media was diluted to treat primary astrocytes with 2 and 0.2 nM concentration of Aβ40 and Aβ42 oligomers, respectively. Astrocytes were exposed to APPSwe neuron‐conditioned media (APPSwe_NCM) or wild type neuron conditioned media (WT_NCM) and the astrocyte‐conditioned media was collected after 24 h. The conditioned media from stimulated astrocytes (APPSwe_ACM) or control (WT_ACM) were subjected to mass spectrometry, together with APPSwe_NCM or WT_NCM (Figure 1a), to control for the components that were already present in neuron media. Importantly, exposure to Aβ‐containing media at the concentration and time used in this study did not result in astrocyte cell death, as we previously reported (Perez‐Nievas et al., 2021), and therefore altered cellular viability is not a confounding factor in the proteomic analysis.

An initial analysis using traditional shotgun proteomics identified 109 proteins, 14 of which were significantly altered in the secretome of astrocytes exposed to APPswe neuron conditioned media (APPSwe_ACM) compared to wild type (WT_ACM) (data not shown). Proteomics of the secretome is usually limited by the high abundance of serum proteins, which creates an excessive dynamic range between albumin and other proteins contained in the media. Our experimental design required the use of serum‐like supplement to ensure neuron survival, and this contains albumin and other proteins in high abundance (Brewer et al., 1993). Albumin immunodepletion strategies were not considered to avoid the nonspecific loss of proteins of interest (Bellei et al., 2011). To overcome these limitations, we prepared an in‐house library of proteins contained in the conditioned media of astrocytes grown in media without serum for 24 h and used it as a matching library following a previously published protocol (Geyer et al., 2016).

We identified 2095 proteins in the secretome of astrocytes treated with media from 5 independent neuronal cultures (Table S1). Figure 1b shows a volcano plot comparing the secretome from astrocytes treated with media from APPswe neurons (APPswe_ACM) with that of astrocytes treated WT neurons (WT_ACM), which rendered a list of 94 significantly altered proteins (Table S1). Because astrocytes were treated with media that contains other neuron‐secreted proteins in addition to Aβ oligomers we excluded those proteins that were found both in APPSwe_NCM or WT_NCM respectively (Table S1). This resulted in a final list of 45 astrocyte‐secreted proteins that were affected by exposure to oligomeric Aβ, being differentially expressed in APPSwe_ACM compared to WT_ACM (Figure 1C). Of these identified proteins, 23 and 22 proteins were significantly increased or decreased, respectively, when astrocytes were treated with media from APPswe neurons containing Aβ oligomers (Table 1). Among these proteins increased, we identified known markers of reactive astrocytes such as APOE, vimentin, and SERPINA3N (Escartin et al., 2021; Zamanian et al., 2012), whose expression increases in astrocytes in human AD brains (Viejo et al., 2022), demonstrating the disease relevance of the proteins identified. Further analysis through ELISA and Western blotting, validated the changes observed in two of these proteins, APOE and PPIB, in astrocyte media (Figure S1).

**TABLE 1:** List of proteins showing significant differences in the secretome of astrocytes treated with WT_NCM versus APPSWE_NCM (*p*‐value <0.05).

Up‐regulated proteins		Log_10_(*p‐*value)*
ACTB	Actin, cytoplasmic 1; Beta‐Actin	1.6266
ACTC1	Actin, alpha cardiac muscle 1	1.8713
ACTG1	Actin, cytoplasmic 2	2.5402
ALDH1L1	Aldehyde dehydrogenase 1 family, member L1	1.6360
APOE	Apolipoprotein E	1.3224
COL1A1	Collagen alpha‐1(I) chain	1.9852
COL1A2	Collagen alpha‐2(I) chain	2.4477
PARVA	Alpha‐parvin	1.7085
PCOLCE	Procollagen C‐endopeptidase enhancer 1	1.4432
PDIA3	Protein disulfide‐isomerase A3	1.7630
PEBP1	Phosphatidylethanolamine‐binding protein 1	1.3937
PPIA	Peptidyl‐prolyl cis‐trans isomerase A	4.3614
PPIB	Peptidyl‐prolyl cis‐trans isomerase B	1.3301
PRDX1	Peroxiredoxin‐1	1.9455
PREP	Prolyl endopeptidase	2.8245
PTGDS	Prostaglandin‐H2 D‐isomerase	1.8604
RPL32	Ribosomal protein L32	1.3087
SEPTIN7	Septin‐7	1.4654
SERPINA3N	Serine protease inhibitor A3N	1.4466
SOD3	Extracellular superoxide dismutase [Cu‐Zn]	1.9752
TPM4	Tropomyosin alpha‐4 chain	1.4215
TP53BP1	Tumor suppressor p53‐binding protein 1	1.5400
VIM	Vimentin	1.4315
Down‐regulated proteins		
BCAN	Brevican core protein	1.8948
CCAR2	Cell cycle and apoptosis regulator protein 2	1.3478
CHCHD3	MICOS complex subunit Mic19	2.6624
CHGA	Chromogranin‐A	1.7527
CTTN	Src substrate cortactin	1.5946
DYSF	Dysferlin	1.4324
H6PD	GDH/6PGL endoplasmic bifunctional protein	1.3870
HNRNPH2	Heterogeneous nuclear ribonucleoprotein H2	1.4309
IRGQ	Immunity‐related GTPase family, Q	1.3891
KRT76	Keratin, type II cytoskeletal 2 oral	1.7513
MPP6	Membrane protein, palmitoylated 6	2.9662
NDUFA2	NADH:Ubiquinone Oxidoreductase Subunit A2	1.4054
PFKP	ATP‐dependent 6‐phosphofructokinase, platelet type	2.1682
PRKAA1	5’‐AMP‐activated protein kinase catalytic subunit alpha‐1	1.3570
RAB9A	Ras‐related protein Rab‐9A	1.3923
RELL1	RELT‐like protein 1	1.9533
RPS7	40S ribosomal protein S7	2.5822
SARNP	SAP domain‐containing ribonucleoprotein	1.3805
SCG5	Neuroendocrine protein 7B2	1.4704
SNX5	Sorting nexin‐5	2.0812
SPOCK2	Testican‐2	2.5676
TNR	Tenascin‐R	1.3355

* Log_10_(*p‐*value) >1.3 is equivalent to *p‐*value <0.05.

### 3.2 Over two‐thirds of Aβ‐modulated astrocyte‐secreted proteins follow non‐conventional secretory pathways

**FIGURE 2:**
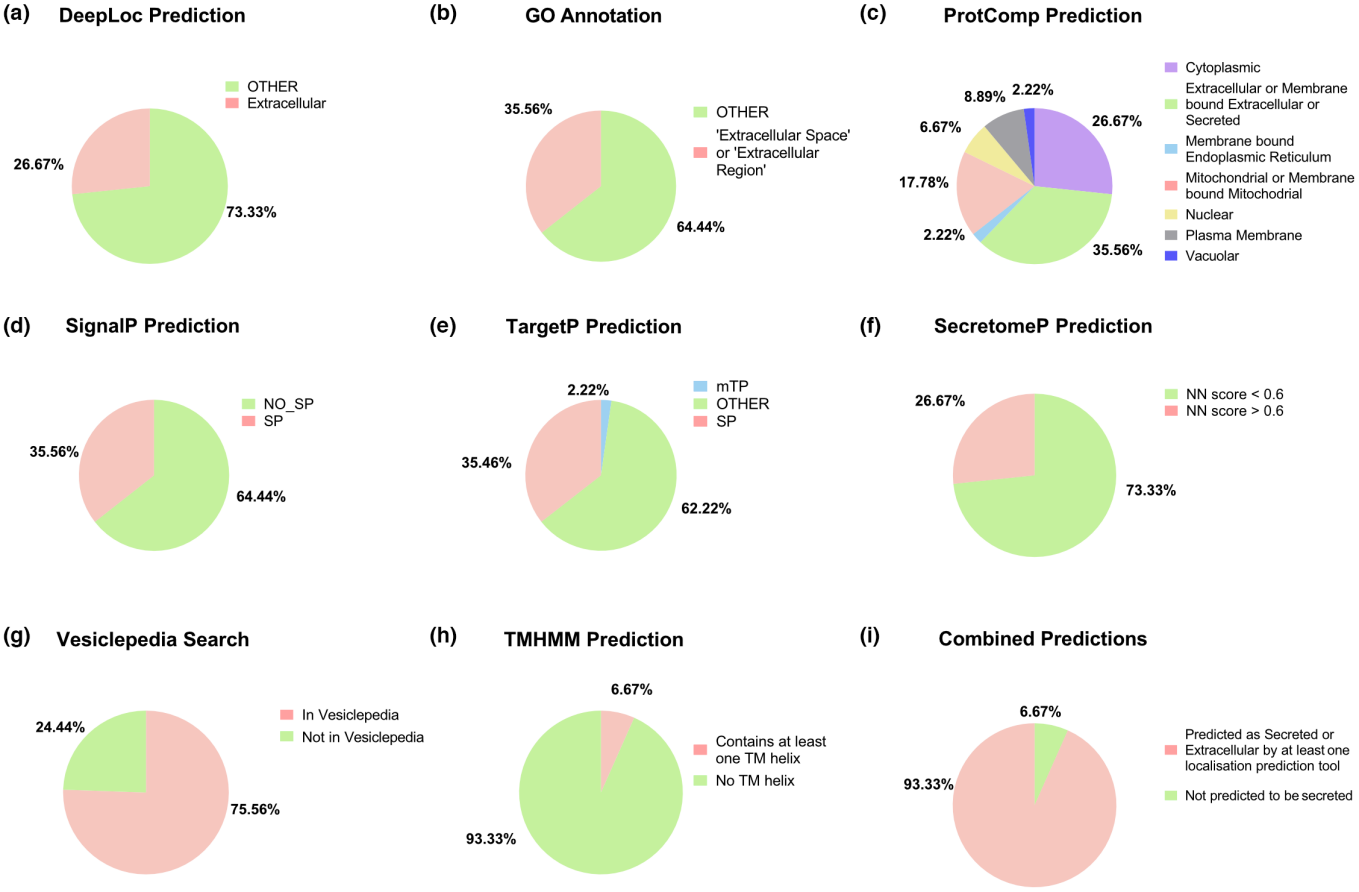
Protein localization and secretion prediction. Different bioinformatics tools (a–h) and combined results (i) were used to predict the localization and route of secretion of proteins identified in ACM. For SecretomeP prediction (f) the NN‐score >0.6 indicates that the mammalian protein is likely to be non‐classically secreted. mTP, mitochondrial transit peptide; NN, non‐classic secretion score; SP, signal peptide; TM, transmembrane.

Prediction tools for subcellular localization based on experimental evidence such as DeepLoc, LAGO terms, or ProtComp, predicted that only 26–35% of the differentially secreted proteins are typically found in the extracellular space (Figure 2a–c). In line with this, 35% of the differentially regulated proteins contained a secretory signal for conventional secretion, while 26% of the proteins were predicted by SecretomeP to be secreted through alternative pathways (Figure 2d–f). According to the Vesiclepedia database (Pathan et al., 2019), 75% of these proteins have been previously identified as being released in extracellular vesicles (EV) (Figure 2g). DeepTMHMM online software (Hallgren et al., 2022) predicted that 3 proteins: dysferlin (DYSF), hexose‐6‐phosphate dehydrogenase (H6PD), and RELT‐like protein 1 (RELL1), contain transmembrane (TM) helices and therefore could be secreted through ectodomain shedding (Figure 2h). Collectively, this combination of eight complementary signal peptide and localization prediction tools, revealed that all proteins, except three, CCAR2, RAB9a, and SARNP, are either predicted to be localized in the extracellular space or to be secreted (Figure 2i and Tables S2 and S3). For those secreted proteins, only one‐third are expected to be released via classical secretory pathways, while most of them will require non‐classical routes, such as release in EVs.

### 3.3 Proteins related to extracellular matrix organization, cytoskeleton, response to oxidative stress, and chaperone functions are overrepresented in the Aβ‐induced astrocyte secretome

**FIGURE 3:**
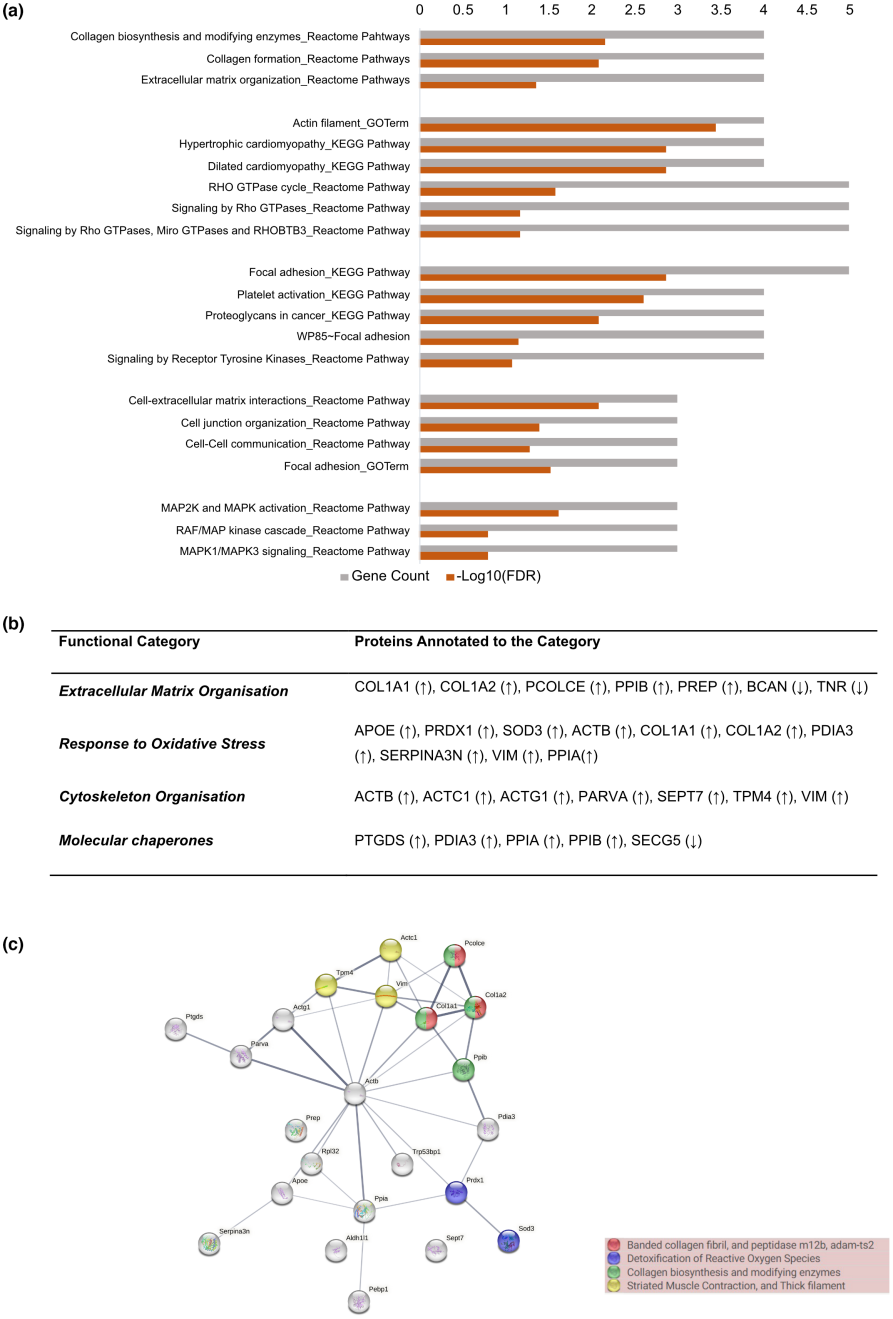
Bioinformatics analysis of proteins differentially secreted in astrocytes treated with Aβ‐oligomers containing media. (a) Functional annotation clustering analysis of differentially upregulated proteins using Database for Annotation, Visualization and Integrated Discovery (DAVID). The analysis included gene ontology (GO) and pathway (KEGG pathway, Reactome, and WikiPathways) terms. Only clusters with an enrichment score of ≥1.3 were considered significant. (b) Table summarizing the functional categories of proteins differentially secreted by Aβ‐treated astrocytes. The categories were chosen and assigned based on the bioinformatics analysis and manual protein annotation. Arrows in the brackets next to the gene names indicate whether the protein was found differentially up‐ (↑) or down‐regulated (↓). (c) Protein–protein interaction network for differentially upregulated proteins generated using STRING. Lines represent protein–protein associations (including but not limited to physical binding) and different thicknesses represent confidence in the interaction. Thicker lines represent higher confidence in the interaction. Circles represent individual proteins and circles of the same color represent proteins that were identified to be involved in the same pathway using STRING local network cluster analysis.

To identify astrocytic pathways and processes that could be affected by alterations in the astrocyte secretome upon exposure to Aβ oligomer containing media, we performed functional annotation clustering using a combination of Gene Ontology terms (Biological Process, Cellular Component, and Molecular Function), Pathways (KEGG, Reactome, and WikiPathways), and manual literature searches. Analysis of the proteins showing increased secretion upon Aβ treatment (Figure 3a,b, Figure S2 and Tables S4–S6) revealed an overrepresentation of proteins involved in the organization of the extracellular matrix (ECM), mostly related to the synthesis of collagen I, the main component of the ECM. Secreted proteins included collagen α‐1 and ‐2, which together make the type I procollagen (Ricard‐Blum, 2011), and enzymes that catalyze the posttranslational modifications necessary to form the collagen triple helix, including the peptidyl‐prolyl isomerase PPIB (Cabral et al., 2014) and the procollagen C proteinase enhancer, PCEP1 or PCOLCE (Adar et al., 1986). PREP, a prolyl endopeptidase involved in the degradation of collagen was also secreted upon Aβ exposure (Gaggar et al., 2008).

Although generally functioning as intracellular proteins, a high proportion of cytoskeletal components were among the proteins found secreted in response to Aβ containing media. These included three out of the six known actin isoforms, β‐ and γ‐cytoplasmic actins (ACTB and ACTG1) and cardiac actin (ACTC1), as well as the actin‐binding proteins tropomyosin α‐4 chain and α‐parvin, septin 7, and the astrocyte‐specific intermediate filament vimentin (Figure 3a,b, Figure S2 and Tables S4–S6). Some of these are important structural components of focal adhesions that mediate contacts with the ECM.

Gene ontology analysis and literature searches also revealed an increase in proteins related to antioxidant activity and in the response to oxygen‐containing compounds among the proteins with increased secretion (Figure 3b, Figure S2, and Tables S5 and S6). Some of these proteins are known to be secreted from astrocytes including SOD3 (Iitsuka et al., 2012; Ioannou et al., 2019), APOE (Kockx et al., 2018; Miyata & Smith, 1996) and SERPINA3N (Sánchez‐Navarro et al., 2021; Wang et al., 2019). While not previously associated with astrocytes, peroxiredoxin‐1 (PRDX1) is an intracellular enzyme with antioxidant activity reported to be secreted by tumor cells (Chang et al., 2005, 2006); and prolyl isomerase (PPIA), although cytoplasmic, is secreted as a defense mechanism in response to oxidative stress (Nigro et al., 2013). The observed changes in collagen type I may also result from a transcriptional response to oxidative stress (Martins et al., 2021).

Similar findings were obtained when using STRING to identify known and predicted physical and functional protein–protein interactions (PPIs) (Szklarczyk et al., 2021), which resulted in a significant PPI enrichment in differentially upregulated proteins (*p‐*value = 3.08 × 10^−11^, *n* = 23), indicating that the number of interactions in these proteins network is significantly different from random networks. STRING local network clustering analysis revealed proteins involved in collagen biosynthesis and detoxification of reactive oxygen species (Figure 3c and Table S7).

A comparison with the 332 proteins comprising the human chaperone (Brehme et al., 2014), identified proteins with reported chaperone activity, representing more than 20% of all proteins showing increased secretion upon treatment with Aβ oligomer containing media (Figure 3b). These included prostaglandin synthase (PTGDS), peptidyl‐prolyl isomerases A, and B (PPIA and PPIB), and protein disulfide isomerase (PDIA3). In addition, further literature searches identified one protein with chaperone activity, secretogranin 5 (SECG5), in the proteins with lowered secretion. Aβ‐chaperone function has been reported for 3 out of 5 of these proteins: PTGDS, PDIA3, and SECG5 (Eninger et al., 2022; Helwig et al., 2013; Kanekiyo et al., 2007; Kannaian et al., 2019), suggesting that astrocytes are a source of extracellular chaperones, whose secretion is likely modulated by exposure to Aβ to protect from its toxicity.

Among the other proteins showing decreased secretion in response to Aβ, Gene Ontology analysis revealed a significant overrepresentation of proteins, such as brevican core protein (BCAN) and tenascin‐R (TNR), that are major components of perineuronal nets, specialized forms of the condensed extracellular matrix that surround groups of neurons and synapses to modulate synaptic plasticity and protect neurons (Reichelt et al., 2019; Wen et al., 2018) (Figure S3a,b and Table S8). Down‐regulated proteins constitute a more heterogeneous group, with no significant clusters found in DAVID and no significant PPI enrichment in STRING (*p‐*value = 0.172, *n* = 22) (Figure S3c).

Altogether, gene ontology and pathway analyses suggest that exposure of astrocytes to Aβ containing medium alters the secretion of proteins involved in extracellular matrix organization and cell‐extracellular matrix interactions, with enhanced secretion of proteins involved in the synthesis of collagen fibers and decreased secretion of components of specialized perineuronal structures in the extracellular matrix. In addition, our study shows an increased secretion of proteins related to the cytoskeleton and response to oxidative stress and proteins with chaperone function.

### 3.4 Comparison of the Aβ‐stimulated astrocyte secretome with astrocyte transcriptomics and CSF proteomics studies in AD

**FIGURE 4:**
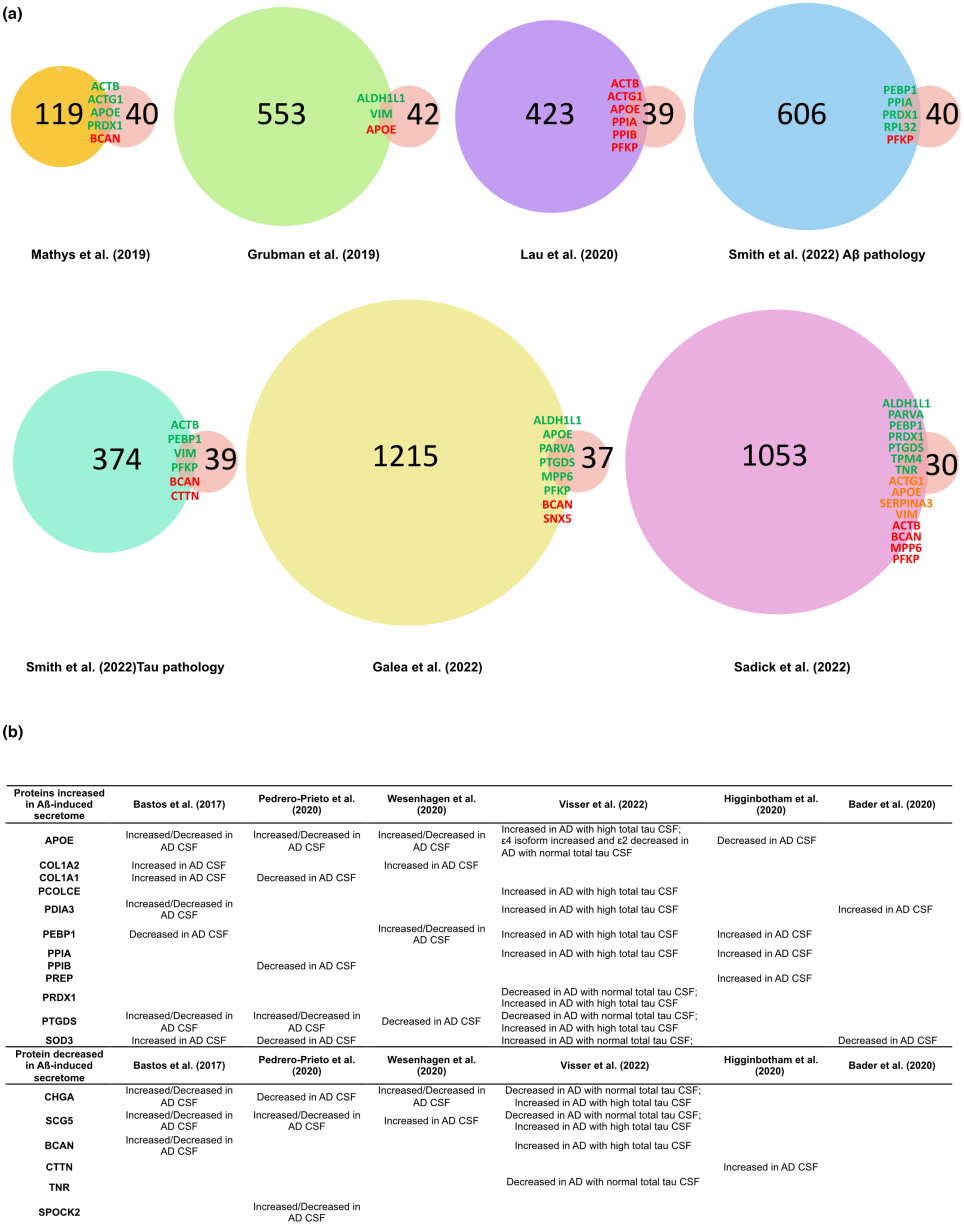
Comparison with transcriptomic and cerebrospinal fluid (CSF) Alzheimer's disease studies. (a) Venn diagrams show the numbers of proteins that overlap between this study and other transcriptomic studies of human AD samples. The number of proteins from transcriptomic studies only includes the proteins that were predicted to be extracellular or secreted using the combination of localization prediction tools used in this study. When changes in gene expression in AD brain compared to controls were in the same direction as the secretion of proteins in response to Aβ oligomers relative to control, names are shown in green; when changes were in the opposite directions, names are shown in red; when genes were reported to be both up‐ and downregulated, the names are shown in orange. (b) Table displaying the proteins that were identified in this study and that were also differentially expressed in CSF from AD compared to controls.

In recent years, single‐nucleus transcriptomics has identified transcriptional changes that occur specifically in astrocytes in the human AD brain. To put our results in context with human data, we compared the list of proteins that we generated with the astrocyte‐specific genes that were found to be differentially expressed in early‐ vs late‐stage human AD brain (Mathys et al., 2019), in AD vs control brain (Grubman et al., 2019; Lau et al., 2020; Sadick et al., 2022) and showing a positive correlation with Aβ or phospho‐tau pathology (Smith et al., 2021). We also included a comparison with a study where astrocyte‐specific gene clusters were applied to 766 whole brain transcriptomes, including control, mild cognitive impairment (MCI), and demented (AD) cases (Galea et al., 2022) (Table S9). After selecting those genes that encode proteins predicted to be secreted, we searched for those that were differentially regulated by Aβ in our study (Figure 4a). Despite the great degree of variability among published RNAseq datasets, some astrocyte‐secreted proteins are consistently found in AD transcriptomic studies. For example, ALDHL1 or PEBP1, whose secretion is increased upon Aβ treatment, were found upregulated in AD in 3 out of 7 studies (Figure 4a). APOE, ACTB, or PFKP are consistently found in nearly all transcriptomic studies, but these were reported as either up‐ or down‐regulated (Figure 4a). BCAN is found in 4 out of 7 comparisons as upregulated in AD, while its secretion from astrocytes is decreased in our study, suggesting a complex role in the regulation of this protein in the AD brain.

To further explore whether changes in protein secretion from astrocytes may reflect changes in CSF in AD, we compared our data with three systematic reviews as well as with three recent proteomic studies, which characterized the proteomic composition of CSF in AD patients compared to controls (Bader et al., 2020;Bastos et al., 2017; Higginbotham et al., 2020; Pedrero‐Prieto et al., 2020; Visser et al., 2022; Wesenhagen et al., 2020) (Figure 4b). Just over half of the proteins showing increased secretion in response to media containing Aβ oligomers (APOE, COL1A1, COL1A2, PCOLCE, PDIA3, PEBP1, PPIA, PPIB, PREP, PREDX1, PTGDS, and SOD3) were identified as altered in AD CSF. A smaller proportion of downregulated proteins, 6 out of 22 proteins (CHGA, SCG5, BCAN, CTTN, TNR, and SPOCK2), appeared as modified in AD CSF in previous studies (Bastos et al., 2017; Higginbotham et al., 2020; Pedrero‐Prieto et al., 2020; Visser et al., 2022; Wesenhagen et al., 2020). For many of the identified proteins, changes are not always consistent across CSF analyses and increased or decreased levels have been simultaneously reported in different studies. APOE is consistently found in nearly all CSF proteomic studies but, similar to the transcriptomic analysis, its levels are found up or downregulated depending on the study. PEBP1, PTGDS, SOD3, CHGA, and SCG5 have been repeatedly identified (4 out of 10 studies) when monitoring for changes in CSF in AD relative to controls (Figure 4b). In fact, chromogranins and secretogranins have been proposed as AD biomarkers with decreased levels of CSF in AD (Abdi et al., 2006; Brinkmalm et al., 2018; Hölttä et al., 2015). Our data suggest that a proportion of the proteins that are commonly found in CSF in AD might have an astrocytic origin.

We used DisGeNET, an online database that compiles information from curated resources, GWAS catalogs, animal models, and published papers (Piñero et al., 2021) to estimate the gene‐disease association (GDA) score for genes encoding differentially secreted proteins in our study. 21 out of 44 (47.7%) genes were found to be associated with AD (Figure S4 and Table S10). Only *APOE* showed a strong association, with a GDA score of 0.7, while the rest of the genes had GDAs ≤0.1, consistent with the notion that, with the exception of *APOE* and *TREM*, most AD‐linked genes individually have only very modest effect sizes (Escott‐Price et al., 2017). While the genes encoding the other proteins have not been formally associated with AD, our data and further comparison with transcriptomic and CSF proteomics studies have identified a new set of proteins that provide insights into the likely functional consequences of astrocyte secretions in AD.

## 4 DISCUSSION

Astrocytes are secretory cells that, in response to brain injury, disease, or signals derived from other cell types, release a myriad of factors that modulate neuronal and non‐neuronal cells and lead to multiple functional responses (Sofroniew, 2020; Verkhratsky et al., 2016). In Alzheimer's disease, astrocytes become reactive, partly due to their exposure to different Aβ species present in the extracellular environment (reviewed in Perez‐Nievas & Serrano‐Pozo, 2018). To our knowledge, only one study has examined the secretory response of astrocytes to Aβ and they used synthetic Aβ42 in the μM range (Lai et al., 2013). Our work sought to understand how the secretory profile of astrocytes is altered in response to naturally occurring soluble oligomeric Aβ, in similar nanomolar concentrations and oligomeric species to those found in AD brain (DaRocha‐Souto et al., 2012; Hudry et al., 2012; Wu et al., 2010). While we cannot exclude additional effects due to other human APP‐derived fragments or to proteins secreted from APPswe expressing neurons, we believe that our experimental setting closely mimics the brain environment to which astrocytes are exposed in AD. We found changes in 45 proteins, of which 23 showed higher secretion and 22 lower secretion upon exposure of astrocytes to a mixture of 1:10 Aβ42:Aβ40. Our study shows some similarities with Lai et al. (2013), with two proteins, APOE and prostaglandin H2 D‐isomerase (PTGDS), identified in both datasets. This suggests a very likely implication of these two proteins in the response to Aβ. In line with this, we found that both proteins are commonly identified in CSF, with PTGDS also known as β‐Trace protein (BTP), one of the main constituents of CSF (Hoffmann et al., 1993; Urade, 2021). The gene‐disease association analysis found APOE to have the strongest link with AD, which is to be expected given that the APOEɛ4 variant is the strongest genetic risk factor for sporadic AD (Serrano‐Pozo et al., 2021). In the brain, APOE is predominantly secreted by astrocytes and, to a lesser extent, by microglia (Serrano‐Pozo et al., 2021). APOE is involved in cholesterol metabolism and, recently, saturated lipids contained in lipoparticles such as APOE have been suggested as mediators of astrocyte‐induced toxicity (Guttenplan et al., 2021). PTGDS catalyzes the conversion of prostaglandin H2 to prostaglandin D2, an arachidonic acid metabolite that modulates the inflammatory response (Joo & Sadikot, 2012) and whose levels are affected in AD brain (Iwamoto et al., 1989; Wong et al., 1992). Moreover, PTGDS‐mediated synthesis of prostaglandin D2 in astrocytes modulates in turn the expression of inflammatory mediators in microglia (Choi et al., 2019) and PTGDS secretion has been reported from oligodendrocytes (Pan et al., 2023) and astrocytes (Giacomelli et al., 1996). Besides these two proteins, our secretory profile differed significantly from that of astrocytes treated with synthetic Aβ42 (Lai et al., 2013). These differences are not unexpected. Unlike synthetic Aβ, which generates oligomers and fibrils easily within minutes to hours, especially when used at high concentrations, naturally secreted Aβ peptides assemble into low molecular weight species (dimers to tetramers). These small soluble oligomeric Aβ species are the most bioactive and have synaptotoxic activity (Shankar et al., 2008; Yang, Li, et al., 2017), and astrocytes treated with these are more likely to reflect their responses in AD brain.

A high proportion of proteins identified in this study are important for the organization of the extracellular matrix (ECM). Alterations in ECM composition have been reported in several neurodegenerative diseases, including AD (Freitas et al., 2021; Ma et al., 2020). In relation to astrocytes, a transcriptomic analysis comparing AD brains with controls identified specific clusters of astrocytes with upregulation of genes involved in ECM organization (Smith et al., 2021). Astrocytes participate in the formation of the extracellular matrix by secreting several factors, including proteoglycans and tenascins (Anwar et al., 2021) and, when reactive or upon Aβ treatment, astrocytes have been implicated in ECM degradation through the release of matrix metalloproteinases (Deb et al., 2003; Muir et al., 2002). Our data also support this active role of astrocytes as modifiers of the ECM composition and function in AD. Of particular interest is the decreased secretion of two of the main components of the perineuronal net (PNN), brevican and tenascin R. PNNs are lattice‐like assemblies of ECM proteins that surround neurons to protect them and modulate neuronal activity (Reichelt et al., 2019; Wen et al., 2018). Evidence from post‐mortem brain studies suggests loss of PNNs in AD (Baig et al., 2005), although other studies found preservation of these structures in disease (Brückner et al., 1999; Lendvai et al., 2013; Morawski et al., 2010). Our data suggest that astrocytes exposed to pathological Aβ may decrease the secretion of PNN components, which can lead to a reduced stabilization of synapses and enhanced exposure to oxidative stress and other toxic molecules, including Aβ itself (Miyata et al., 2007; Suttkus et al., 2012).

Many of the up‐regulated proteins found in this study have antioxidant activity and/or are involved in the response to oxygen‐containing compounds. Sings of oxidative damage are observed in aged and AD brains (Wang et al., 2014), starting in early phases of the disease (Butterfield et al., 2006; Keller et al., 2005). Some of the proteins we found dysregulated in this study have been previously linked to oxidative stress and AD. Peroxiredoxin‐1, which catalyzes the reduction of hydrogen peroxide and similar compounds, was found up‐regulated in the cortex of AD patients (Szeliga, 2020), and a recent large‐scale proteomic study discovered that peroxiredoxin‐1 levels were elevated in both AD brain tissues and CSF (Johnson et al., 2020). Moreover, peroxiredoxin‐1 conferred resistance to Aβ toxicity in PC12 cells, SH‐SY5Y cells, and rat primary hippocampal neurons (Cimini et al., 2013; Cumming et al., 2007). Extracellular superoxide dismutase 3 (SOD3) is another antioxidant enzyme (Wang et al., 2018) that ameliorates the oxidative damage exerted by Aβ25‐35 in SH‐SY5Y (Yang, Wei, et al., 2017), and its levels in CSF are frequently changed in AD (Bader et al., 2020; Perrin et al., 2011; Ringman et al., 2012; Visser et al., 2022). In cultured astrocytes, Aβ triggers the production of reactive oxygen species (ROS) (Abramov et al., 2004; Askarova et al., 2011). The astrocyte response we report here may represent a self‐defense mechanism to protect themselves against the oxidative stress caused by Aβ or a mechanism to provide antioxidant support to neurons (Jiwaji & Hardingham, 2022).

We also report an increased secretion of molecular chaperones. While typically considered intracellular proteins, chaperones can be secreted through classical and non‐classical secretion mechanisms with important roles in extracellular proteostasis (Chaplot et al., 2020). PTGDS is found in plaques in AD brain and its Aβ‐chaperone activity has been reported, through both inhibiting Aβ aggregation and promoting fibril disaggregation (Kanekiyo et al., 2007; Kannaian et al., 2019). PDIA3 is a disulfide isomerase with a thiol oxidoreductase activity that promotes protein folding in the ER (Chichiarelli et al., 2022). It was detected in human CSF bound to Aβ (Erickson et al., 2005) and, more recently, Di Risola et al. have reported that treating SHSY5Y neuroblastoma cells with synthetic Aβ led to increased extracellular PDIA3, which can bind to Aβ 25–35 in vitro to reduce aggregation and counteract its cellular toxicity (Di Risola et al., 2022). While not previously included within the chaperone network (Brehme et al., 2014), secretogranin 5, also known as 7B2, whose levels are reduced in our Aβ‐exposed astrocyte secretome, has also been suggested to function as a chaperone, since it colocalizes with Aβ plaques in human AD brain and prevents fibrillation of Aβ40 and Aβ42 in vitro and blocks their cytotoxic effect in culture (Helwig et al., 2013). Single‐cell RNAseq and immunohistochemical studies have frequently reported that genes involved in proteostasis and chaperone‐mediated responses are up‐regulated specifically in astrocytes in AD (Grubman et al., 2019; Lau et al., 2020; A. M. Smith et al., 2021; Viejo et al., 2022) which, together with our study, may imply that astrocytes are a source of extracellular chaperones to protect the disease brain environment.

Three ubiquitously expressed actin isoforms are among the secreted proteins in response to Aβ. Different roles of actin as an extracellular protein have been reported (Sudakov et al., 2017), including its function as a “danger‐associated molecular pattern” (DAMP) (Ahrens et al., 2012; Srinivasan et al., 2016). DAMPs trigger an inflammatory response by binding to receptors such as Toll‐like receptors, which are expressed mostly in microglia and astrocytes in the brain (Sofroniew, 2020). Other proteins found in our study have been suggested as DAMPs, including peroxiredoxin 1 (PRDX1), which binds to TLR‐4 after secretion from cancer cells (Liu et al., 2016; Riddell et al., 2010), or prolyl endopeptidase (PREP) that cleaves collagen to produce proline‐glycine‐proline (PGP), which acts as a DAMP (Patel & Snelgrove, 2018) and participates in the recruitment of neutrophils (Weathington et al., 2006). In addition, other proteins upregulated in the Aβ‐induced astrocyte secretome have been directly implicated in inflammation. PTGDS synthesizes prostaglandin D2, which functions as a modulator of the inflammatory response (Joo & Sadikot, 2012); peptidylprolyl isomerase A (PPIA) is mainly cytoplasmic but it can also be secreted in response to different inflammatory stimuli such as LPS (Hoffmann & Schiene‐Fischer, 2014), and inhibition of extracellular PPIA reduces neurotoxicity and neuroinflammation markers such as NF‐kB activation in a mouse model of amyotrophic lateral sclerosis (ALS) (Pasetto et al., 2017). Altogether, a large proportion of the secreted proteins identified in response to Aβ oligomers may be involved in further amplifying or diminishing the inflammatory response initiated by Aβ. Surprisingly, we did not identify any cytokines or chemokines. Cytokines and chemokines are typically secreted from astrocytes, and their secretion profile is altered in the AD brain and more specifically upon treatment with Aβ (González‐Reyes et al., 2017), including our own studies using a similar experimental setting (Perez‐Nievas, 2021). Cytokine and chemokine secretion is typically detected by immunoassay, while its detection through mass spectrometry is challenging due to their low molecular weight and low abundance relative to other proteins in the media (Kupcova Skalnikova et al., 2017), which may explain their absence in this dataset.

Proteins showing reduced secretion in this study constitute a more heterogeneous group. Among these proteins, we identified proteins related to mitochondrial integrity and function (NDUFAD and CHCHD3), glucose metabolism (H6PD and PFKP), energy sensors (PRKAA1 and SARNP), RNA binding proteins (HNRNPH2), intracellular trafficking (RAB9A and SNX5), as well as secreted neuroendocrine peptides (CHGA and SCG5).

Astrocyte proteins with increased secretion in response to Aβ largely overlapped with proteomics studies conducted in AD CSF. Increased levels of astrocytic proteins in CSF, such as GFAP, have been associated with Aβ deposition, levels of phospho/total tau, and with markers of synaptic dysfunction (Milà‐Alomà et al., 2020; Salvadó et al., 2021). In fact, a CSF proteomic study in the APPPS1 mouse model of amyloidosis highlighted increased levels of proteins related to glial activation and identified APOE and SERPINA3 as CSF biomarkers (Eninger et al., 2022). Our data suggest that some of the proteins that are commonly found in CSF in AD, might be of astrocytic origin and that changes in astrocytes can be reflected in the CSF. This may be the case for some proteins already proposed as AD biomarkers such as chromogranins (Abdi et al., 2006; Brinkmalm et al., 2018; Hölttä et al., 2015).

To conclude, our study shows that in response to human Aβ oligomers, in similar concentrations and species to those found in AD brain, astrocytes present a distinct profile encompassing ECM molecules, antioxidant proteins, components of the cytoskeleton, chaperones, and regulators of inflammatory responses. These changes mimic some of those identified in AD transcriptomic and CSF studies and contribute to a better understanding of how astrocytes play a pivotal role in the molecular response to Aβ in AD and can be relevant for the identification of novel biomarkers.

## AUTHOR CONTRIBUTIONS

VM performed the mass spectrometry analysis and database search; AG did the bioinformatic analysis and comparison with transcriptomic and CSF studies; FY validated the proteomic data; BGP‐N and MJ‐S undertook the animal and cell culture work and prepared samples for analysis; VM and AB conceived and designed the proteomics analysis; BGP‐N and MJ‐S conceived and designed the animal and cell work and the overall project.

## CONFLICT OF INTEREST STATEMENT

The authors declare no conflict of interest to declare that are relevant to the content of this article.

### OPEN RESEARCH BADGES

This article has earned an Open Data badge for making publicly available the digitally‐shareable data necessary to reproduce the reported results. The data is available at: www.ebi.ac.uk/pride/archive/projects/PXD036343

## Supporting information


Supplementary Tables.

Figure S1.
Figure S2.
Figure S3.
Figure S4.

## Data Availability

The data that support the findings of this study are openly available ProteomeXchange Consortium via the PRIDE (Perez‐Riverol et al., 2022) partner repository with the dataset identifier PXD036343.
