# Supplementary material for: Proteomics of the astrocyte secretome reveals changes in their response to soluble oligomeric Aβ
Source: J Neurochem. 2023 Jun 11;166(2):346–66. doi: 10.1111/jnc.15875 (PMC10952722; doi:10.1111/jnc.15875)
Supplement: Supplementary file 2 — Figure S1. Figure S2. Figure S3. Figure S4. [file JNC-166-346-s002.zip › Supplementary Figure 1-4.pdf]

Proteomics of the astrocyte secretome reveals changes in their response to soluble oligomeric Aß

Vittoria Matafora[1,*], Alena Gorb [2,*], Fangjia Yang[2,*], Wendy Noble[2], Angela Bachi [1,#], Beatriz Gomez Perez-Nievas [2,#], Maria Jimenez-Sanchez [2,#]

1. IFOM ETS- The AIRC Institute of Molecular Oncology, Via Adamello 16, 16039 Milan, Italy.
2 Department of Basic and Clinical Neuroscience, Maurice Wohl Clinical Neuroscience Institute, Institute of Psychiatry, Psychology and Neuroscience, King's College London, 5 Cutcombe Road, London, SE5 9RX, UK

*Joint first authors
[#]Corresponding authors: maria.jimenez_sanchez@kcl.ac.uk, beatriz.gomez_perez-nievas@kc.ac.uk, angela.bachi@ifom.eu

- Supplementary Figures 1-4

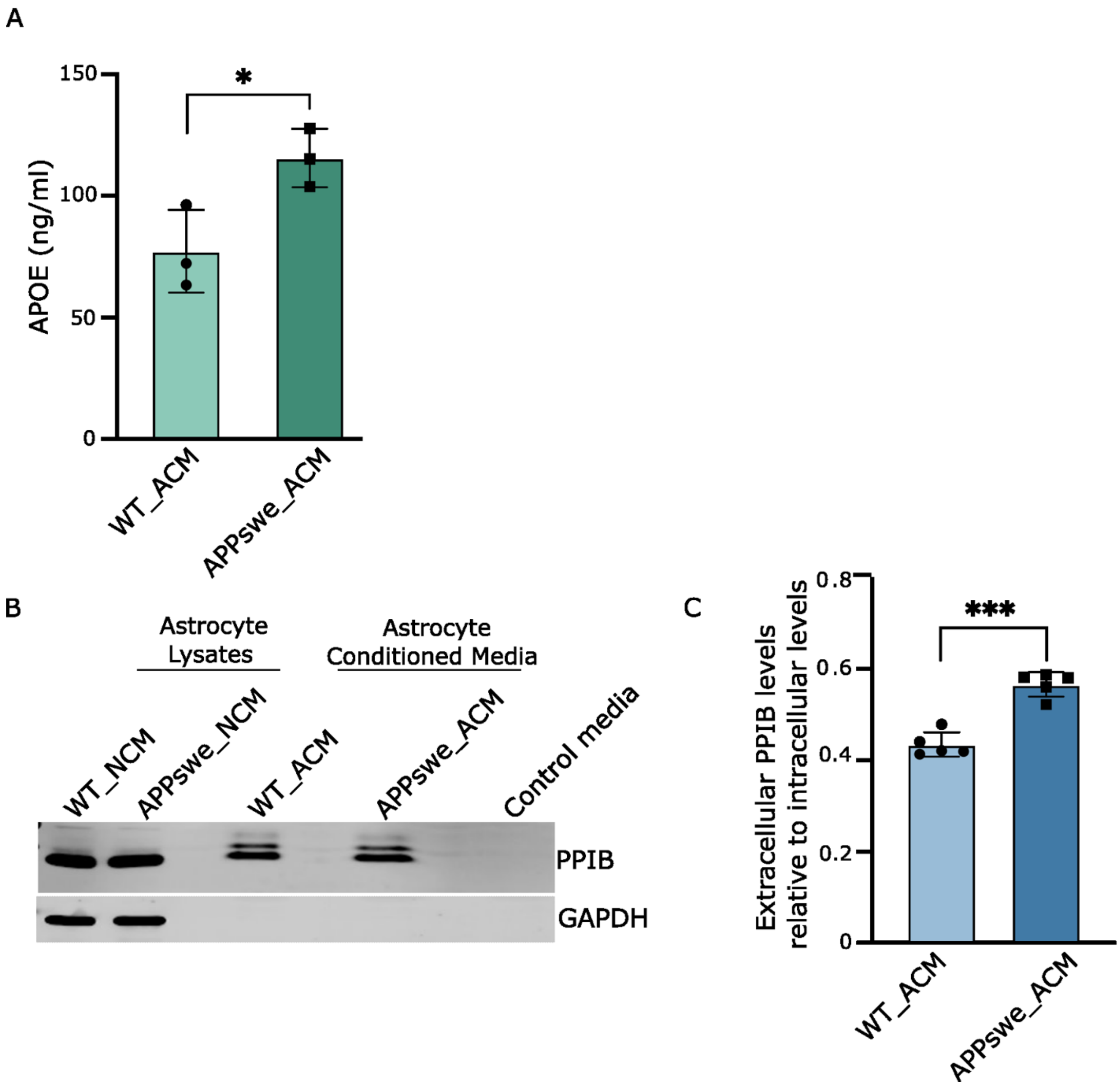

Supplementary Figure 1. Validation of targets identified by proteomics. (A) Levels of APOE in conditioned media (ACM) from astrocytes treated with either WT or APPswe neuronal media were detected using an ELISA assay. Graph shows mean of 3 biological replicates with error bars representing standard deviation. **(B)** The levels of PPIB were detected in lysates or ACM of astrocytes treated with either WT or APPswe neuronal media. Media only was included to discard any unspecific band present in the serum. (**C)** Graph shows the ratio of PPIB levels in ACM relative to lysates from 5 biological replicates, with error bars representing standard deviation. Unpaired Student's t-test (* $p<0.05$, *** $p<0.001$).

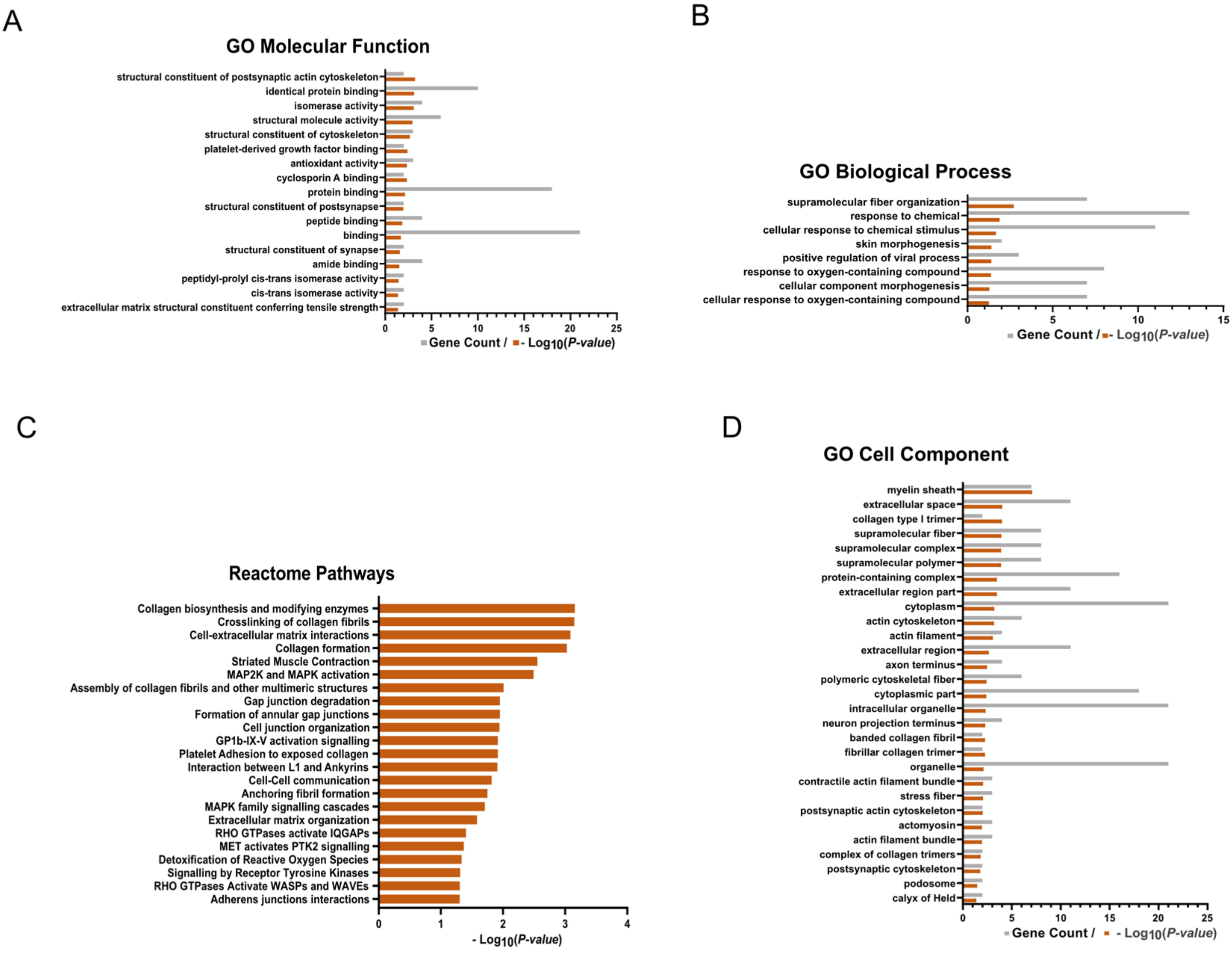

Supplementary Figure 2. Gene Ontology (GO) analysis for Molecular Function (**A**), Biological Process (**B**), Cell Component (**C**), or Reactome pathway analysis (D) of proteins differentially upregulated in the media of astrocytes treated with media containing Aβ oligomers. The overrepresentation significance threshold was set at the *p*-value < 0.05.

A

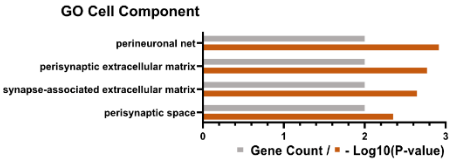

B

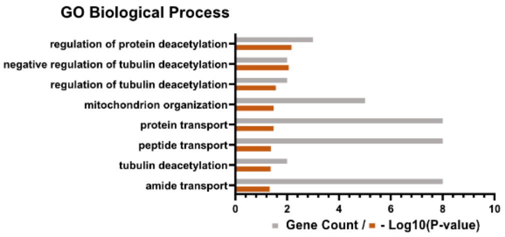

C

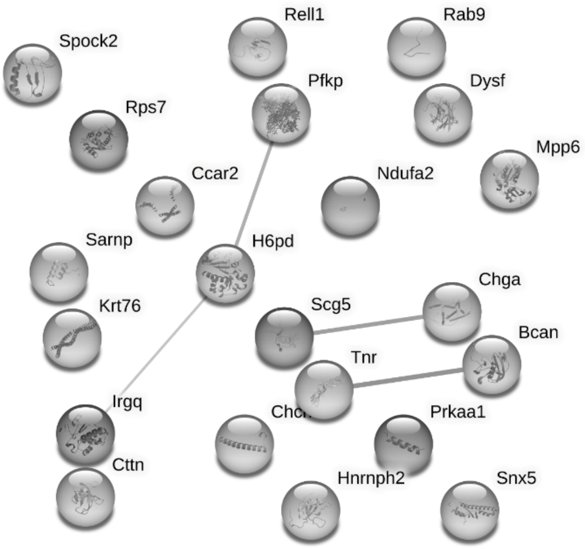

Supplementary Figure 3. Gene Ontology (GO) analysis for Cell Component (**A**) and Biological Process (**B**) of proteins differentially upregulated in the media of astrocytes treated with media containing Aß oligomers. The overrepresentation significance threshold was set at the *p*-value < 0.05. (**C**) Protein-protein interaction network for differentially downregulated proteins generated using STRING. Lines represent protein-protein associations (including but not limited to physical binding) and different thicknesses represent confidence in the interaction. Thicker lines represent higher confidence in the interaction. Circles represent individual proteins.

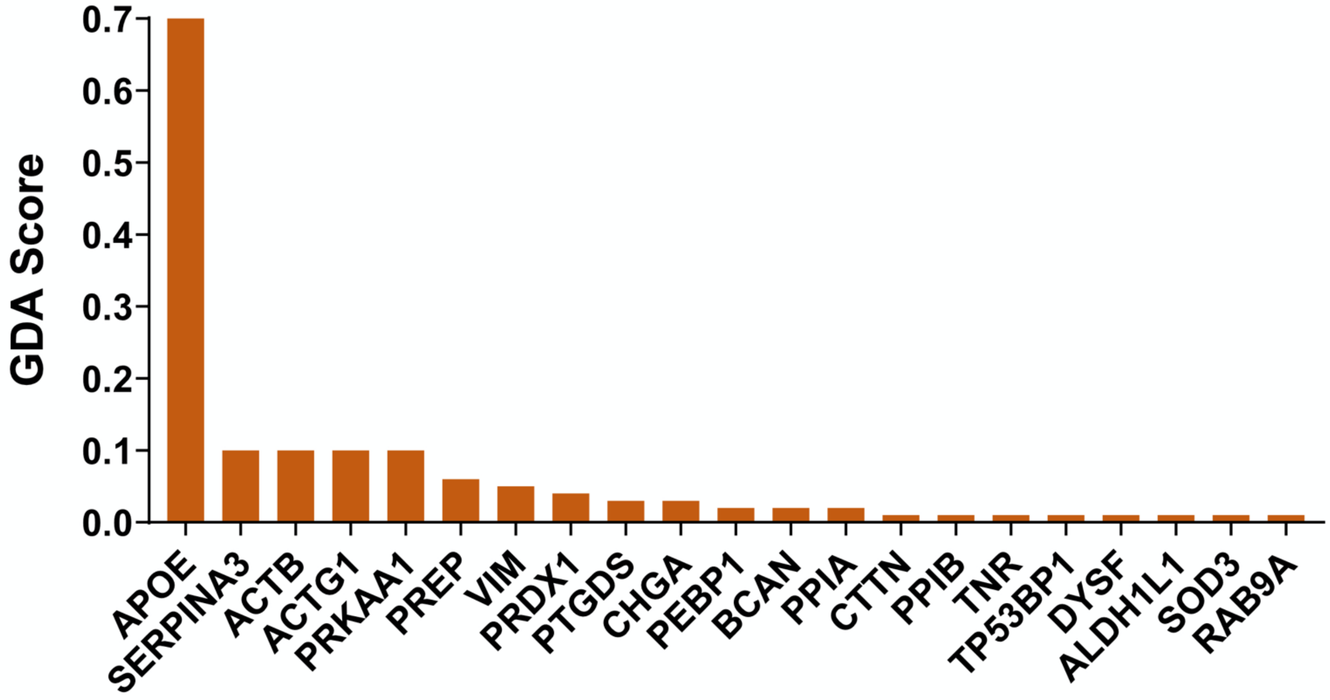

Supplementary Figure 4. Gene-disease association (GDA) scores for the genes coding for proteins identified in this study and found associated with AD using the DisGeNET database. Score shows its association that were found to be associated with Alzheimer's disease.
